# Broad‐Spectrum Antibiotics Attenuate the Chemotherapeutic Efficacy of Doxorubicin in MDA‐MB‐468 Breast Cancer Cells

**DOI:** 10.1155/ijbc/9772651

**Published:** 2026-07-26

**Authors:** Benedict Broni Boadi, Kwadwo Fosu, Elizabeth Besiwa Quaw, Kezia Yaa Awortwe, Anthony Eremondsere Agholo, Bernadine Tuah, Jude Tetteh Quarshie, Abiola Isawumi, Kwadwo Asamoah Kusi, Anastasia Rosebud Aikins

**Affiliations:** ^1^ West African Centre for Cell Biology of Infectious Pathogens (WACCBIP), University of Ghana, Accra, Ghana, ug.edu.gh; ^2^ Department of Biochemistry, Cell and Molecular Biology, University of Ghana, Accra, Ghana, ug.edu.gh; ^3^ Department of Immunology, Noguchi Memorial Institute for Medical Research, College of Health Sciences, University of Ghana, Accra, Ghana, ug.edu.gh

**Keywords:** antibiotics, breast cancer, chemotherapy, doxorubicin, inflammation

## Abstract

**Background:**

Breast cancer remains a global health challenge due to rising incidence, chemoresistance, and tumor relapse. In cancer management, antibiotics are often used to treat bacterial infections in immunocompromised patients. However, the direct impact of antibiotics on cancer progression and treatment outcomes is not well understood. This study investigated the effects of four antibiotics—ciprofloxacin, amoxicillin‐clavulanic acid, tetracycline, and meropenem—on breast cancer cells, both alone and in combination with the chemotherapeutic agent doxorubicin (DOX).

**Methods:**

Using the MDA‐MB‐468 breast cancer cell line, we assessed cell viability using the MTT assay. The impact of antibiotics on apoptosis was assessed using Annexin V/PI flow cytometry. Additionally, we evaluated the expression of stem cell markers CD133, CD44, and CD24 posttreatment using flow cytometry. Finally, the Luminex assay was used to measure the cytokine and chemokine release by the cells.

**Results:**

All of the antibiotics reduced cell viability in a time‐ and dose‐dependent manner but reduced DOX‐induced cytotoxicity. The antibiotics also induced early apoptosis in the cells but significantly reduced DOX‐induced apoptosis, supporting our earlier observation that these antibiotics diminish the efficacy of DOX chemotherapy. Furthermore, amoxicillin‐clavulanic acid, ciprofloxacin, and meropenem diminished the efficacy of DOX chemotherapy by promoting stemness in cancer cells. The antibiotics exhibited variable effects on cytokine and chemokine production.

**Conclusion:**

Although these antibiotics exhibit anticancer activity, their coadministration with DOX may reduce its therapeutic efficacy and promote cancer stemness. These findings highlight the need for further investigation into antibiotic–chemotherapy interactions in breast cancer treatment.

## 1. Introduction

Breast cancer remains the leading cause of morbidity and mortality among women worldwide [1]. Although its incidence has seen a significant increase over the years, the rate of breast cancer‐related mortality is gradually reducing, owing to targeted interventions addressing modifiable risk factors, improving healthcare, and access to better treatment [1]. Nevertheless, therapeutic resistance and toxicity of available drugs limit their usefulness, resulting in treatment failure, progression to metastatic disease, and even worse symptoms in patients [2]. This is especially worse in areas like sub‐Saharan Africa, where access to healthcare and targeted therapy is still limited [3].

Breast cancer is classified into four molecular subtypes based on the expression of estrogen receptor (ER), progesterone receptor (PR), and human epidermal growth factor receptor 2 (HER2). These subtypes include luminal A (ER+ and/or PR+, and HER2‐), luminal B (ER+ and/or PR+, and HER2+), HER2 (ER−, PR−, and HER2+), and triple‐negative breast cancer (ER−, PR−, and HER2−) [4]. The molecular subtypes inform clinicians on what drugs should be used for treatment, for example, HER2 inhibitors are used for the treatment of HER2+ breast cancers. Regardless of the subtype, breast cancer patients are given chemotherapy solely or in combination with other agents [2].

Currently, the most effective chemotherapeutic drug for breast cancer is doxorubicin (DOX), a member of the anthracycline family [5]. It mediates its anticancer effect by intercalating into DNA and stabilizing the topoisomerase II‐DNA complex, resulting in DNA double‐strand breaks. This eventually leads to the generation of free radicals, mitochondrial dysfunction, and apoptosis [6]. Although DOX has shown great benefits in breast cancer treatment, its clinical use is limited by myelotoxicity, gonadotoxicity, and cardiotoxicity [7]. Furthermore, DOX may cause immunosuppression due to off‐target toxicity on immune cells [8].

Immunosuppression in breast cancer patients is associated with worse prognosis and poor survival [9]. Immunosuppression due to cytotoxic chemotherapies also increases the risk of bacterial infections like *S. aureus*, *S. agalactiae*, *S. pneumoniae,* and *Enterococcus* species [10]. The risk of bacterial infection further increases in cancer patients who have had surgery, stem cell transplantation, or are experiencing radiation‐related neutropenia [11]. Indeed, infections are thought to at least contribute to the deaths of approximately 50% of patients with hematological malignancies or solid tumors [11]. Due to this, antibiotics are indispensable in the prevention or treatment of bacterial infections in cancer patients.

Evidence shows that some antibiotics by themselves possess anticancer properties. For example, ciprofloxacin (CIP), a bacterial DNA gyrase and topoisomerase IV inhibitor, has been shown to inhibit the proliferation of colon carcinoma cell lines [12], nonsmall‐cell lung cancer cell lines [13], and prostate cancer cell lines [14]. Another antibiotic—meropenem (MER), a *β*‐lactam that inhibits bacterial cell wall synthesis—has been demonstrated to reduce the viability of SKOV3 ovarian cancer cell lines when loaded on graphene oxide‐gelatin [15]. Furthermore, tetracyclines (TETs) such as demeclocycline, minocycline, and doxycycline have been reported to enhance antitumor T‐cell immunity [16]. Although these findings support the use of antibiotics in cancer treatment, a study of triple‐negative breast cancer patients found that antimicrobial prescription is associated with lower overall and breast cancer‐specific survival, regardless of neutrophil count and disease severity [17]. Another study concluded that administering antibiotics to end‐stage cancer patients does more harm than good due to toxic side effects [18]. Additionally, studies have reported negative effects of antibiotic exposure on immune checkpoint inhibitor therapy in nonsmall cell lung cancer, renal cell carcinoma, urothelial carcinoma, and melanoma [19]. This highlights the need to evaluate the impact of antibiotics on cancer treatment and disease outcomes.

In this study, we aimed to assess the impact of amoxicillin‐clavulanic acid (AMX), CIP, MER, and TET on the viability, apoptosis, stemness, and cytokine secretion of breast cancer cells, administered both as monotherapies and in combination with DOX. Specifically, we used the MDA‐MB‐4688 cell line as our model as it is a well‐characterized TNBC cell line and closely recapitulates the disease [20]. We show that these broad‐spectrum antibiotics attenuate the chemotherapeutic efficacy of DOX in MD Anderson Metastatic Breast‐468 (MDA‐MB‐468) breast cancer cells.

## 2. Materials and Methods

### 2.1. Cell Lines and Culture

MDA‐MB‐468 cell line (PRID: CVCL_0419), obtained from the American Type Culture Collection, was maintained in Dulbecco′s Modified Eagle′s Medium (DMEM) supplemented with 10% fetal bovine serum (FBS) and 1% penicillin‐streptomycin‐glutamine (all purchased from Gibco‐Life Technologies, Carlsbad, California, United States) at 37°C in a humidified atmosphere containing 5% CO_2_.

### 2.2. Compounds/Drugs

DOX (D1515), AMX (AABH97CE769F), CIP (17850), MER (1392454), and TET (87128) were purchased from Sigma‐Aldrich, St. Louis, Missouri, United States. The compounds were dissolved in dimethyl sulfoxide (DMSO) as instructed by the manufacturer.

### 2.3. Cell Viability Assay

The effects of the compounds on cell viability were determined using an MTT assay, method adopted from [21]. Briefly, the cells were seeded into 96‐well plates at a density of 1 × 10^4^ cells/well and incubated at 37°C for 24 h. They were treated with DOX, AMX, CIP, MER, or TET for 24, 48, and 72 h. Then 20 *μ*L of 2.5 mg/mL 3‐(4,5‐dimethylthiazol‐2‐yl)‐2,5‐diphenyltetrazolium bromide (MTT) (Sigma‐Aldrich, St. Louis, Missouri, United States) was added to each well and incubated at 37°C for 4 h. Afterward, 100 *μ*L of acidified isopropanol was added to each well and incubated at 37°C for 30 min. Absorbance was read at 570 nm with a Varioskan LUX multimode microplate reader (Thermo Fisher Scientific, Carlsbad, California, United States). From the absorbance values, percent cell viabilities were calculated.

### 2.4. Cell Treatments

For all monotherapies, the cells were treated with the 24‐h IC50 of compounds, that is, DOX (0.18 *μ*M), AMX (0.24 *μ*M), CIP (0.17 *μ*M), MER (0.20 *μ*M), or TET (0.12 *μ*M). For all cotreatments, the cells were treated with the 72‐h IC50 of compounds, that is, AMX (0.17 *μ*M), CIP (0.06 *μ*M), MER (0.05 *μ*M), or TET (0.06 *μ*M) in combination with DOX (0.10 *μ*M). We used low doses of antibiotics and DOX for cotreatment experiments to ensure that our observations did not result from toxic off‐target effects which typically occur at high drug concentrations [22, 23].

### 2.5. Flow Cytometry

MDA‐MB‐468 cells were either untreated or treated with DOX or antibiotics for 24 h. The cells were collected, prepared, and stained with antibodies according to the manufacturer‐recommended guidelines. Cells were stained with fluorescein isothiocyanate (FITC)‐labeled Annexin‐V (BD Biosciences, Cat# 556419, RRID: AB_2665412) and propidium iodide (BD Biosciences, Cat# 556463, RRID: AB_2869075) to assess apoptosis. The cancer stem cell population was determined by staining cells with antibodies against the cancer stem‐like markers CD44 (BD Biosciences, Cat# 555478, RRID: AB_395870), CD24 (BD Biosciences, Cat# 555428, RRID: AB_395822), and CD133 (BD Biosciences, Cat# 566596, RRID: AB_2744280). The stained cells were analyzed using a FACScan flow cytometer (BD Biosciences, San Jose, California, United States). The data generated were collected and processed using FlowJo 10.10.0 software (BD Life Sciences).

### 2.6. Luminex Assay

Cells were seeded at a density of 5 × 10^5^ and incubated at 37°C for 24 h to attach. The following day, the cells were treated with drugs for 48 h. Then, cell culture supernatants were collected and prepared for cytokine profiling. The levels of the cytokines IL‐2, IL‐4, IL‐6, IL‐8, IL‐10, CCL2, CCL5, CXCL1, CXCL2, TNF‐alpha, IL‐1 beta, and IFN‐gamma were measured using a commercially available 12‐plex cytokine panel (R&D Systems, Minneapolis, MN, USA). The Luminex MAGPX 200 platform was used to determine the levels of cytokines. The assays included a series of known concentrations to generate standard curves, ensuring accurate quantification. The data generated were collected and processed using Bio‐Plex Manager 6.0 software (Bio‐Rad, Irvine, California, United States).

### 2.7. Statistical Analysis

GraphPad Prism 9.1.2 (GraphPad Software, San Diego, United States) was used for all statistical analyses. One‐way analysis of variance followed by Dunnett′s post hoc test was used to compare differences between multiple groups. Data are presented as the mean ± standard error of the mean (SEM) of three independent experiments. Group differences were considered statistically significant when *p* < 0.05.

## 3. Results

### 3.1. Selected Antibiotics Reduce MDA‐MB‐468 Viability but Are Antagonistic to DOX

**Figure 1:**
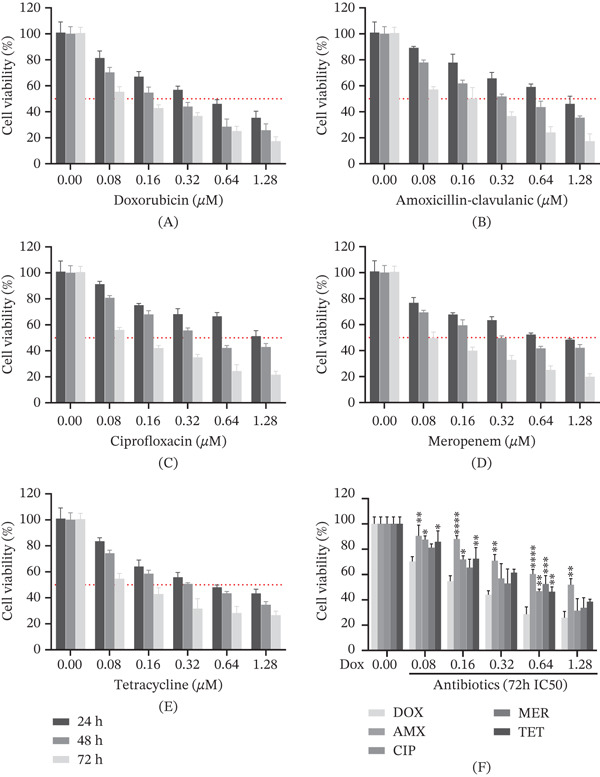
Selected antibiotics reduce MDA‐MB‐468 viability but are antagonistic to doxorubicin. Cells were treated with increasing concentrations of (A) doxorubicin, (B) amoxicillin‐clavulanic acid, (C) ciprofloxacin, (D) meropenem, (E) tetracycline for 24, 48, and 72 h, or (F) increasing concentrations of doxorubicin in combination with the 72‐h IC50 of the various antibiotics for 48 h. Data represent mean ± SEM of three independent experiments performed in triplicate.  ^∗^ *p* ≤ 0.1,  ^∗∗^ *p* ≤ 0.01,  ^∗∗∗^ *p* ≤ 0.001,  ^∗∗∗∗^ *p* ≤ 0.0001 versus DOX.

Chemotherapy often results in immunosuppression, increasing the risk of infection and thereby necessitating the frequent administration of antibiotics to cancer patients [10]. Although some antibiotics exhibit anticancer properties [24], whether they affect chemotherapy outcomes remains an active area of research. To address this, we investigated whether the antibiotics used in our study could affect the viability of MDA‐MB‐468 breast cancer cells. Results from MTT assays showed that all the antibiotics tested reduced cell viability in a dose‐ and time‐dependent manner (Figure 1A–E). Although AMX showed lower potency than DOX, the other antibiotics (CIP, MER, and TET) demonstrated higher potencies (Table 1).

**Table 1:** IC50 values for treatment conditions.

Time (h)	IC50 (*μ*M) of compounds				
	DOX	AMX	CIP	MER	TET
24	0.18	0.24	0.17	0.20	0.12
48	0.16	0.13	0.16	0.09	0.12
72	0.10	0.17	0.06	0.05	0.06

Abbreviations: AMX, amoxicillin‐clavulanic; CIP, ciprofloxacin; Dox, doxorubicin; MER, meropenem; TET, tetracycline.

To further evaluate the impact of these antibiotics on DOX′s efficacy, we conducted combination treatments using antibiotic–DOX dual therapy. Unexpectedly, we observed a marked reduction in DOX‐induced cytotoxicity at all concentrations. Notably, cotreatment with AMX led to an unexpected increase in cell viability (Figure 1F). These findings suggest that the combination of DOX and certain antibiotics may attenuate its chemotherapeutic efficacy, an outcome that could potentially compromise cancer treatment.

### 3.2. Selected Antibiotics Exert Their Anticancer Effect via Apoptosis, but Inhibit DOX‐Induced Late Apoptosis

**Figure 2:**
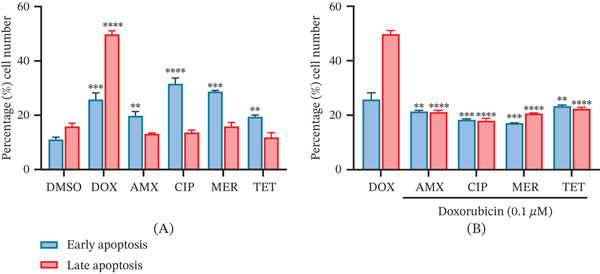
Selected antibiotics exert their anticancer effect via apoptosis but inhibit doxorubicin‐induced late apoptosis. MDA‐MB‐468 cells were treated (A) singly with doxorubicin or antibiotics (B) with doxorubicin in combination with antibiotics for 24 h. Data represent mean ± SEM of three independent experiments performed in triplicate.  ^∗∗^ *p* ≤ 0.01,  ^∗∗∗^ *p* ≤ 0.001,  ^∗∗∗∗^ *p* ≤ 0.0001 versus DMSO in mono therapy or versus DOX in dual therapy.

Apoptosis is the primary mechanism by which several chemotherapeutic agents mediate their action. Indeed, drugs like DOX have been demonstrated to induce apoptosis via activation of caspases and disruption of mitochondrial membrane potential ([25]). Additionally, some antibiotics have been shown to induce apoptosis in B‐cell lymphoma [26] and osteosarcoma cells [27]. Given this, we investigated whether the antibiotics used in our study exert their anticancer activity through the induction of apoptosis. Apoptosis was assessed using Annexin V‐FITC/PI staining following antibiotic treatment. We found that all the antibiotics tested induced early apoptosis in the cells. Interestingly, a subset of cells treated with DOX progressed to late apoptosis, which may reflect its greater potency or a distinct mechanism of action (Figure 2A, Figure S1). We further determined the apoptotic effect of antibiotic‐DOX cotreatment. As shown in Figure 2B (Figure S2), the presence of antibiotics significantly reduced DOX‐induced apoptosis, supporting our earlier observation that these antibiotics diminish the efficacy of DOX chemotherapy.

### 3.3. Antibiotic Monotherapy and Antibiotic‐DOX Dual Therapy Have Opposing Effects on CD133/CD44 Stem Cell Marker Expression

**Figure 3:**
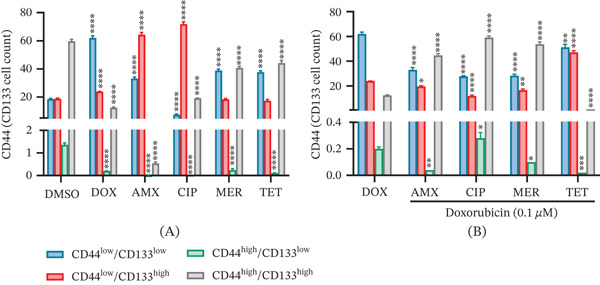
Antibiotic monotherapy and antibiotic‐doxorubicin dual therapy have opposing effects on CD133/CD44 stem cell marker expression. MDA‐MB‐468 cells were treated (A) singly with doxorubicin or antibiotics (B) with doxorubicin in combination with antibiotics for 24 h. Data represent mean ± SEM of three independent experiments performed in triplicate.  ^∗∗^ *p* ≤ 0.05,  ^∗∗^ *p* ≤ 0.01,  ^∗∗∗^ *p* ≤ 0.001,  ^∗∗∗∗^ *p* ≤ 0.0001 versus DMSO in mono therapy or versus DOX in dual therapy.

Stem cell‐like properties of cancer cells are key contributors to drug resistance and tumor recurrence [28]. To determine whether the selected antibiotics could influence stemness, we assessed their impact on the expression of stem cell markers CD133 and CD44. CD44 (cluster of differentiation 44) and CD133 (cluster of differentiation 133) are cell surface glycoproteins in maintaining cell membrane architecture, cell–cell interactions, and cell adhesion and migration. High expression of these markers (CD133^high^/CD44^high^) is associated with stem‐like properties, such as self‐renewal, tumor initiation, drug resistance, and metastasis [29, 30]. As expected for MDA‐MB‐468 cells, untreated cells exhibited a low proportion of CD133^low^/CD44^low^ expressing cells and a high proportion of CD133^high^/CD44^high^ expressing cells. We also observed an expected increase in CD133^low^/CD44^low^ population and a decrease in CD133^high^/CD44^high^ population in the DOX‐treated cells. This indicates that DOX reduces the classic predominant highly tumorigenic cancer stem cell population in MDA‐MB‐468 cells and increases the population of differentiated, nonstem‐like, therapy‐sensitive cells. Antibiotic treatment modulated stem cell marker expression to varying degrees. Notably, we observed a general increase in CD133^low^/CD44^low^ population, accompanied by a reduction in CD133^high^/CD44^high^ population following antibiotic monotherapy. Additionally, AMX and CIP treatment induced a marked increase in CD133^low^CD44^high^ population, with AMX also causing a pronounced decrease in CD133^high^/CD44^high^ expressing cells (Figure 3A, Figure S3).

Given that the cotreatment reduced DOX efficacy (Figure 1F), we next investigated whether the cotreatment also altered the expression of stem cell markers. Surprisingly, all antibiotics except TET reduced the CD133^low^/CD44^low^ population and increased the CD133^high^/CD44^high^ population when combined with DOX (Figure 3B, Figure S4). These findings suggest that when given in combination with DOX, AMX, CIP, and MER promote stem cell‐like properties in MDA‐MB‐468 cells, whereas TET may counteract this effect.

### 3.4. Antibiotic Monotherapy and Antibiotic‐DOX Dual Therapy Have Opposing Effects on CD44/CD24 Stem Cell Marker Expression

**Figure 4:**
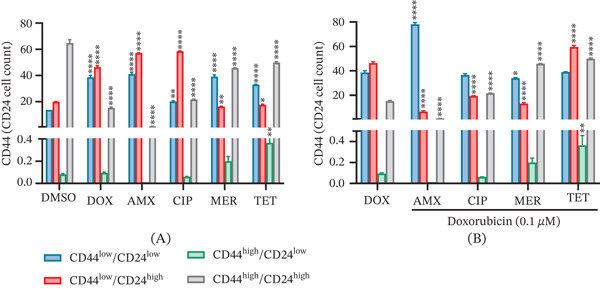
Antibiotic monotherapy and antibiotic‐doxorubicin dual therapy have opposing effects on CD44/CD24 stem cell marker expression. MDA‐MB‐468 cells were treated (A) singly with doxorubicin or antibiotics (B) with doxorubicin in combination with antibiotics for 24 h. Data represent mean ± SEM of three independent experiments performed in triplicate.  ^∗∗^ *p* ≤ 0.05,  ^∗∗^ *p* ≤ 0.01,  ^∗∗∗^ *p* ≤ 0.001,  ^∗∗∗∗^ *p* ≤ 0.0001 versus DMSO in mono therapy or versus DOX in dual therapy.

To further confirm the effect of the antibiotics on stem cell‐like properties, we analyzed the expression of additional stem cell markers CD44 and CD24. Just like CD133 and CD44, CD24 (cluster of differentiation 24) is a cell surface protein involved in cell adhesion and metastasis. In contrast to their expression in stem cells, low or absent CD24 (CD24^low^) is associated with less differentiated stem cell‐like states [31]. Treatment with antibiotics led to an increase in the CD44^low^/CD24^high^ population (Figure 4A), which is indicative of a more differentiated, nonstem‐like phenotype, therapy‐sensitive breast cancer cells. In contrast, there was no significant change in the CD44^high^/CD24^low^ population, except in the TET‐treated group, which showed an increase (Figure 4A, Figure S5). Since the CD44^high^/CD24^low^ is the canonical breast cancer stem cell phenotype associated with tumor initiation, metastasis, and chemoresistance ([32]), our observation suggests that TET may increase the population of breast cancer stem cells.

In the context of combination therapy, AMX, CIP, and MER reduced the CD44^low^/CD24^high^ population, further confirming their potential to induce stem cell‐like characteristics when used alongside DOX. Conversely, TET increased the population of CD44^low^/CD24^high^ cells, validating its ability to reduce stem cell‐like characteristics in the presence of DOX (Figure 4B, Figure S6). Collectively, our findings indicate that AMX, CIP, and MER may compromise the efficacy of DOX chemotherapy by promoting stemness in cancer cells, whereas TET may enhance DOX chemotherapy.

### 3.5. Antibiotics Exhibited Differential Effects on Cytokine and Chemokine Production

**Figure 5:**
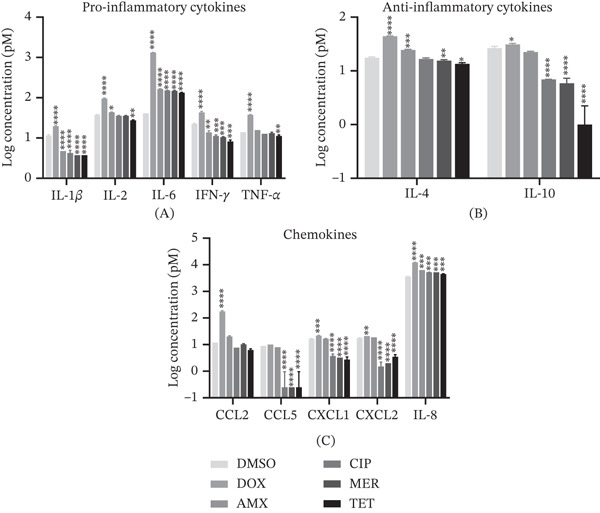
Antibiotics exhibited differential effects on cytokine and chemokine production. Levels of (A) proinflammatory cytokines, (B) anti‐inflammatory cytokines, and (C) chemokines produced by MDA‐MB‐468 cells treated with doxorubicin or selected antibiotics for 48 h. Data represent mean ± SEM of three independent experiments performed in triplicate.  ^∗^ *p* ≤ 0.05,  ^∗∗^ *p* ≤ 0.01,  ^∗∗∗^ *p* ≤ 0.001,  ^∗∗∗∗^ *p* ≤ 0.0001 versus DMSO.

DOX has been reported to increase inflammatory responses in cancer cells, animal models, and cancer patients [6, 33]. Thus, we investigated whether the antibiotics, by themselves, also promote or diminish the production of proinflammatory and anti‐inflammatory cytokines and chemokines in the cancer cells. We observed an expected increase in proinflammatory cytokine production in DOX‐treated cells. Generally, the selected antibiotics reduced proinflammatory cytokine production, except for IL‐6, which was significantly increased following treatment (Figure 4A). We also observed moderate increases in the levels of anti‐inflammatory cytokines IL‐4 and IL‐10 following DOX treatment. On the other hand, the selected antibiotics had little to no effect on IL‐4 levels, whereas IL‐10 levels were significantly reduced by CIP, MER, and TET treatment (Figure 4B). Concerning chemokine production, we observed notable variations across treatments and cell lines. For example, CCL2, CXCL1, CXCL2, and IL‐8 levels were significantly elevated in DOX‐treated cells, whereas CCL5 levels were unaffected. Moreover, CIP, MER, and TET reduced the levels of CCL5, CXCL1, and CXCL2 while increasing the levels of IL‐8 (Figure 5C).

## 4. Discussion

Cancer patients are frequently treated with antibiotics due to their increased susceptibility to bacterial infections resulting from chemotherapy‐induced immunosuppression [34]. Nonetheless, the impact of antibiotics on chemotherapy is still not delineated, primarily due to limited understanding of the effect of antibiotics on cancer cells and treatment outcomes [19]. This study examined the effects of four commonly used antibiotics—CIP, AMX, TET, and the last‐resort antibiotic MER—on MDA‐MB‐468 breast cancer cells. We also investigated how these antibiotics influence the efficacy of DOX; a standard chemotherapeutic agent used in breast cancer treatment.

Our results revealed that all four antibiotics exhibited cytotoxic effects against MDA‐MB‐468 cells in a time‐ and dose‐dependent manner, indicating their potential to limit breast cancer cell growth. This finding is supported by another research study, which showed that TET and ampicillin induced a dose‐ and time‐dependent cytotoxic effect in HT‐29 colorectal cancer cells [35]. Additionally, it has been reported that beta‐lactam antibiotics like AMX and MER selectively target tumor cells and exhibit anticancer properties [36].

Surprisingly, the combination of DOX and these antibiotics attenuated its chemotherapeutic efficacy, suggesting potential antagonistic effects. This is consistent with the findings of a previous study where in SCC‐15 cells, AMX in combination with cisplatin reduced the cytotoxic impact of cisplatin by up to 21.8% [37]. Another study reported that ceftazidime and cefepime antagonize 5‐fluorouracil′s effect in colon cancer cells [38]. Furthermore, a clinical study of 4290 patients with metastatic renal cell carcinoma demonstrated that antibiotic use was associated with worse outcomes in patients treated with immunotherapy‐based regimens [39]. This observation was also seen in advanced renal cell and nonsmall‐cell lung cancer patients treated with immune checkpoint inhibitors and was attributed to antibiotic‐mediated gut dysbiosis through alteration of gut microbiota diversity and composition [40]. Although our research does not extend to animal models or human patients, our results suggest that the antibiotics promote cancer survival, at least to an extent, independent of the gut microbiome. On the other hand, there have been studies that reported contrary findings. For example, MER was demonstrated to enhance the efficacy of 5‐fluorouracil [38]. Again, CIP was found to increase paclitaxel‐induced cytotoxicity in ABCB1‐resistant cancer cells [41], which contrasts our data showing that CIP and MER reduce the efficacy of DOX in MDA‐MB‐468 cells. This suggests that antagonist, additive or synergistic effects of antibiotic‐anticancer drug combination treatment are cell‐type and drug type‐specific and need further research.

In this study, DOX, CIP, AMX, and MER induced apoptosis in these cells, suggesting a shared ability to trigger cancer cell death. Of all the drugs used in this study, DOX caused a significant subset of cells to progress to the late apoptotic stage. This could be attributed to its apoptosis‐inducing properties and distinct mechanisms of action such as interposing DNA base pairs and halting protein synthesis [6]. Upon cotreatment, however, the antibiotics inhibited DOX‐induced late apoptosis. A recent study demonstrated that ceftazidime and cefepime significantly antagonized 5‐fluorouracil‐induced S‐phase arrest and apoptosis induction in DLD‐1 and HCT‐116 cells [38]. Our study and theirs highlight the potential of antibiotics to impact chemotherapy outcomes. The antiapoptotic effects following combination treatment could be attributed to several mechanisms, including antibiotic‐mediated stabilization of mitochondrial function, suppression of caspase activation, or redirection of cell fate toward survival pathways such as autophagy or senescence. Although we did not investigate the exact mechanism by which the antibiotics inhibited late DOX‐induced apoptosis, our findings suggest that certain antibiotics may antagonize the proapoptotic effects of DOX, potentially reducing its therapeutic efficacy.

CSCs play a critical role in tumor growth and therapeutic resistance, making them key targets in cancer drug development. Indeed, there is active research aimed at identifying druggable vulnerabilities in CSCs‐dependent pathways [42]. In our study, we observed a reduction in CD44^high^/CD133^high^ and CD44^high^/CD133^low^ populations after treatment with all the antibiotics. This suggests an intrinsic ability of the antibiotics to suppress cancer stem cell‐like properties as CD133 and CD44 are cancer stem cell markers associated with tumor initiation, self‐renewal, migration, and resistance to therapy [43, 44]. Notably, treatment with AMX, MER, and TET increased the CD44^low^/CD133^low^ population. This shift may reflect the differentiation of CD44^high^/CD133^high^ and CD44^high^/CD133^low^ cells into less stem‐like populations, which are more susceptible to conventional chemotherapeutic agents. These findings were confirmed by the increase in CD44^low^/CD24^high^ population following AMX and CIP treatment, indicating that they induce a more differentiated, nonstem cell‐like phenotype, therapy‐sensitive breast cancer cells. In contrast, MER and TET increased the CD44^high^/CD24^low^ cells, a subset associated with invasive behavior and resistance to anticancer drugs [45]. The increment in this population following antibiotic treatment may indicate a potential risk of promoting tumor invasion and drug resistance. As such, our findings reinforce the possibility that CIP, TET, AMX, and MER may exhibit antitumor or protumor effects through stem cell‐like cancer cell populations.

Cytokines are key components of the tumor microenvironment. Chronic inflammation and cytokine secretion create immunosuppressive environments that promote tumor progression and therapeutic resistance [46, 47]. For instance, increased secretion of IL‐6, IL‐4, and IL‐8 by cancer cells promotes angiogenesis, metastasis, and tumor growth [48]. From our results DOX induced the production of proinflammatory cytokines, as has been previously reported by our group [33]. In contrast, the antibiotics seemed to reduce the production of proinflammatory cytokines IL‐1*β*, IL‐2, IFN‐*γ*, and TNF‐*α*. We also observed reduced IL‐10 by CIP, MER and TET treatment. Given the role of IL‐10 in promoting cancer drug resistance [49], this reduction could indicate the potential of these antibiotics to mitigate drug resistance mechanisms although these observations are not conclusive. Moreover, DOX treatment significantly elevated the levels of CCL2, CXCL1, CXCL2 and IL‐8 levels the antibiotics CIP, MER and TET reduced their levels. Although these observations are not conclusive, we show that antibiotics have differential effects on cancer cells.

In conclusion, our study showed that cotreatment with DOX and antibiotics reduced the chemotherapeutic efficacy of DOX. This was evident in decreased apoptosis, reduced cytotoxicity, and increased cancer stem cell populations compared with DOX monotherapy. These results suggest potential drug–drug interactions that could undermine therapeutic outcomes, highlighting a possible antagonistic relationship between DOX and certain antibiotics. This finding has important clinical implications, especially for cancer patients receiving concurrent chemotherapy and antibiotic treatment and warrants further investigation to guide antibiotic use in oncology settings.

## Author Contributions

Conceptualization: B.B.B., K.F., and A.R.A.; methodology: B.B.B., E.B.Q., A.E.A., and K.Y.A.; formal analysis: B.B.B., E.B.Q., K.F., and J.T.Q.; writing—original draft Preparation: B.B.B., A.E.A., and E.B.Q.; writing—review and editing: J.T.Q., B.T., K.A.K., A.I., A.R.A.; supervision: K.A.K., A.I., and A.R.A.

## Funding

This work was supported by a DELTAS Africa grant (DEL‐22‐014: Awandare). This research was funded in whole or in part by the Science for Africa Foundation to the Developing Excellence in Leadership, Training and Science in Africa (DELTAS Africa) programme [DEL‐22‐014] with support from Wellcome and the UK Foreign, Commonwealth & Development Office and is part of the EDCPT2 programme supported by the European Union. For purposes of open access, the author has applied a CC BY public copyright license to any author accepted manuscript version arising from this submission.

## Conflicts of Interest

The authors declare no conflicts of interest.

## Supporting information


**Supporting Information** Additional supporting information can be found online in the Supporting Information section. Figure S1: “Selected Antibiotics Induce Apoptosis in MDA‐MB‐468 cells.” Figure S2: “Selected Antibiotics Reduce Late Apoptosis of Doxorubicin on MDA‐MB‐468 cells.” Figure S3: “Antibiotics Single Treatments Reduce CD44/CD133 Cancer Stem Cell populations in MDA‐MB‐468 breast cancer cells.” Figure S4: “Doxorubicin‐antibiotic show higher CD44/CD133 cancer stem cell populations in MDA‐MB‐468 breast cancer cells.” Figure S5: “Antibiotics Single Treatments Reduce CD24/CD44 cancer stem cell populations in MDA‐MB‐468 breast cancer cells.” Figure S6: “Doxorubicin‐Antibiotics Treatments Show Higher on CD24/CD44 cancer stem cell population in MDA‐MB‐468 breast cancer cells.”

## Data Availability

The data that support the findings of this study are available from the corresponding authors upon reasonable request.
