# Supplementary material for: Broad‐Spectrum Antibiotics Attenuate the Chemotherapeutic Efficacy of Doxorubicin in MDA‐MB‐468 Breast Cancer Cells
Source: Int J Breast Cancer. 2026 Jul 26;2026:9772651. doi: 10.1155/ijbc/9772651 (PMC13402892; doi:10.1155/ijbc/9772651)
Supplement: Supplementary file 1 — Supporting Information Additional supporting information can be found online in the Supporting Information section. Figure S1: “Selected Antibiotics Induce Apoptosis in MDA‐MB‐468 cells.” Figure S2: “Selected Antibiotics Reduce Late Apoptosis of Doxorubicin on MDA‐MB‐468 cells.” Figure S3: “Antibiotics Single Treatments Reduce CD44/CD133 Cancer Stem Cell populations in MDA‐MB‐468 breast cancer cells.” Figure S4: “Doxorubicin‐antibiotic show higher CD44/CD133 cancer stem cell populations in MDA‐MB‐468 breast cancer cells.” Figure S5: “Antibiotics Single Treatments Reduce CD24/CD44 cancer stem cell populations in MDA‐MB‐468 breast cancer cells.” Figure S6: “Doxorubicin‐Antibiotics Treatments Show Higher on CD24/CD44 cancer stem cell population in MDA‐MB‐468 breast cancer cells.” [file IJBC-2026-9772651-s001.docx]

**Supplementary Figures**

**Supplementary Figure 1 – “Selected antibiotics induce apoptosis in MDA-MB-468 Breast Cancer Cells”**


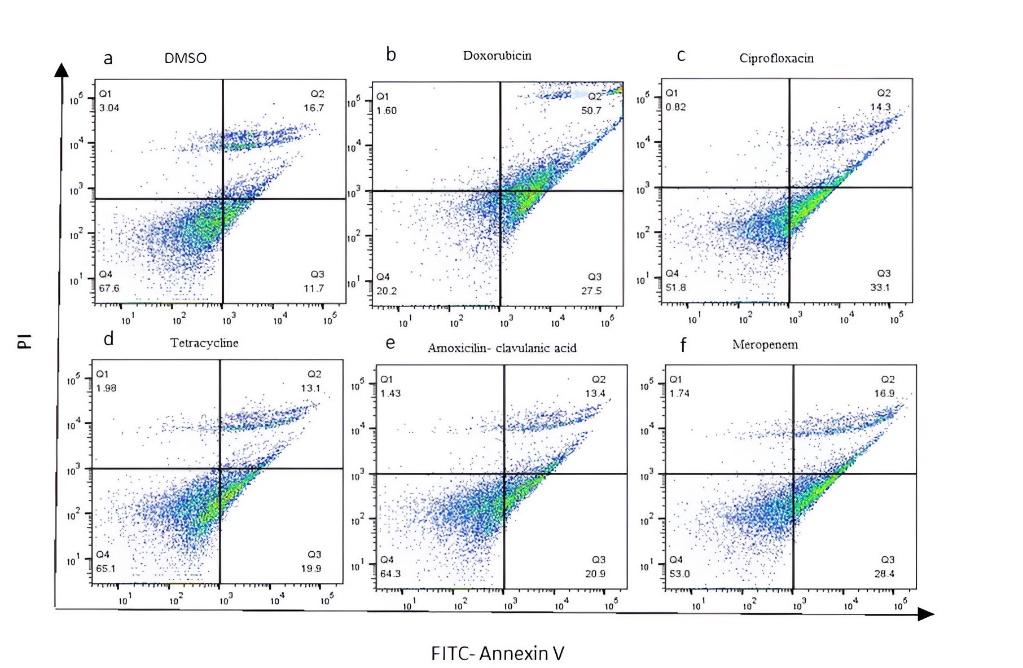


**Figure 1**. Effects of antibiotics on apoptosis of MDA-MB-468 cells a) DMSO, cells treated with b) doxorubicin, c) ciprofloxacin, d) tetracycline e) amoxicillin-clavulanic acid, and f) meropenem. Q1: PI+/AV-, Q2: PI+/AV+, Q3: PI-/AV+, Q4: PI-/AV- populations.

**Supplementary Figure 2 – “Selected Antibiotics Reduce Late Apoptosis of Doxorubicin on MDA-MB-468 cells”**


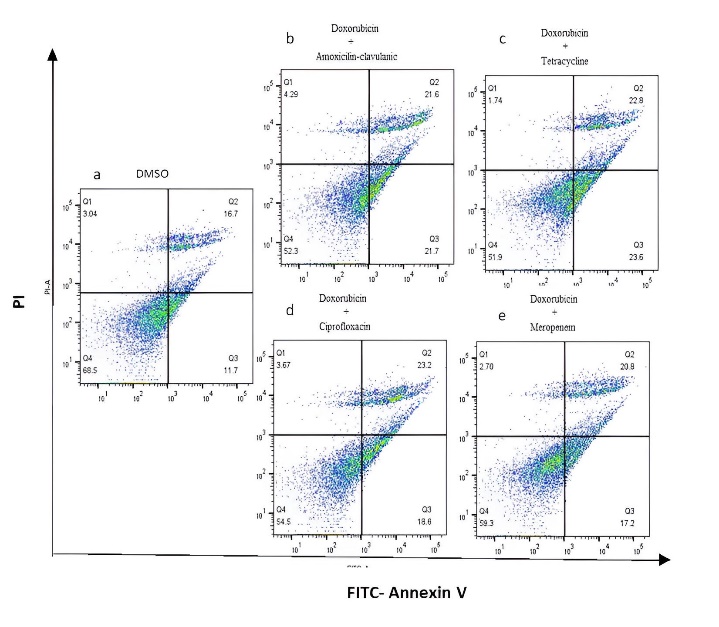


Figure 2. Effects of doxorubicin-antibiotic treatment on apoptosis a) DMSO b) doxorubicin + amoxicillin-clavulanic acid, c) doxorubicin + tetracycline, d) doxorubicin + ciprofloxacin, and e) doxorubicin + meropenem. Q1: PI^+^/AV^-^, Q2: PI^+^/AV^+^, Q3: PI^-^/AV^+^, Q4: PI^-^/AV^-^ populations

**Supplementary Figure 3 – “Antibiotics Single Treatments Reduce CD44/CD133 Cancer Stem Cell populations in MDA-MB-468 breast cancer cells”**


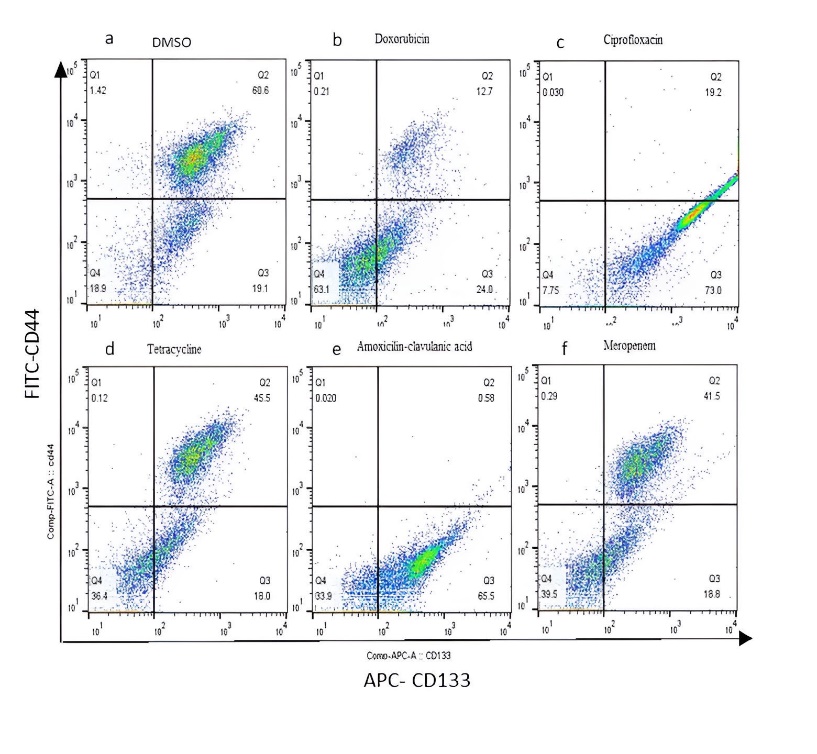


**Figure 3.** Effects of antibiotics on CD133/CD44 stem cancer stem cell populations in MDA-MB-468 breast cancer cells. a) DMSO b) doxorubicin, c) ciprofloxacin, d) amoxicillin-clavulanic acid, e) tetracycline, and f) meropenem. Q1: CD44high/CD133low, Q2: CD44high/CD133high, Q3: CD44low/CD133high, Q4: CD44low/CD133low populations

**Supplementary Figure 4 – “Doxorubicin-antibiotic show higher CD44/CD133 cancer stem cell populations in MDA-MB-468 breast cancer cells”**


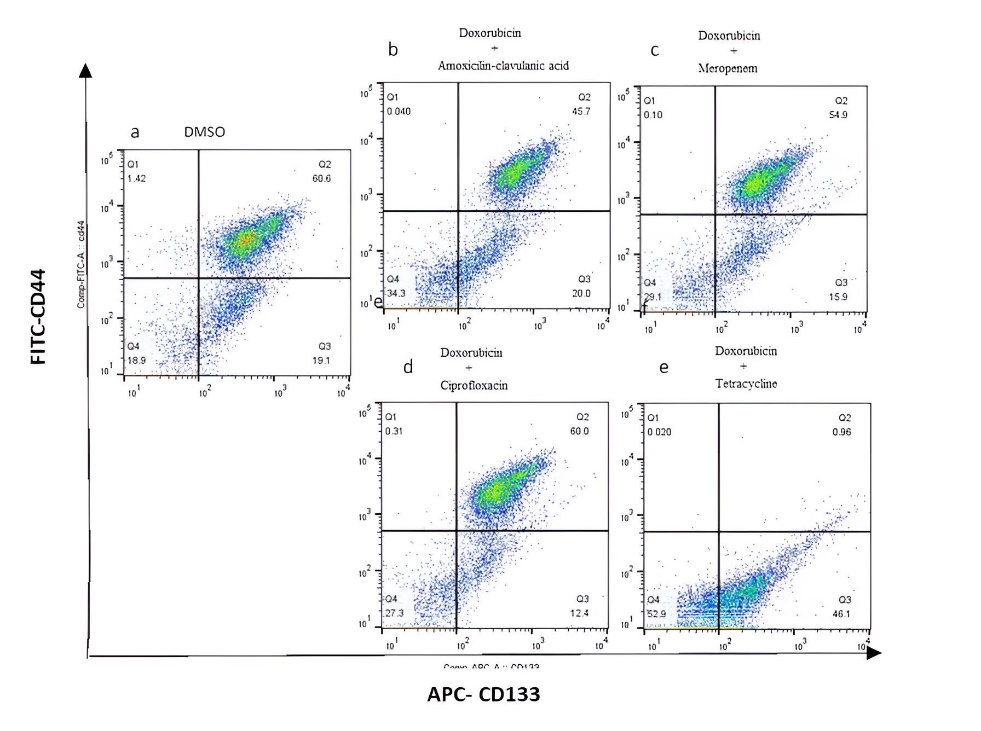


**Figure 4**. Effects of co-treating doxorubicin combined with antibiotics CD44 and CD133 cancer stem cell populations. a) DMSO b) doxorubicin + amoxicillin-clavulanic acid, c) doxorubicin + tetracycline, d) doxorubicin + ciprofloxacin, and e) doxorubicin + meropenem . Q1: CD44high/CD133low, Q2: CD44high/CD133high, Q3: CD44low/CD133high, Q4: CD44low/CD133low populations.

**Supplementary Figure 5 – “Antibiotics Single Treatments Reduce CD24/CD44 cancer stem cell populations in MDA-MB-468 breast cancer cells”**


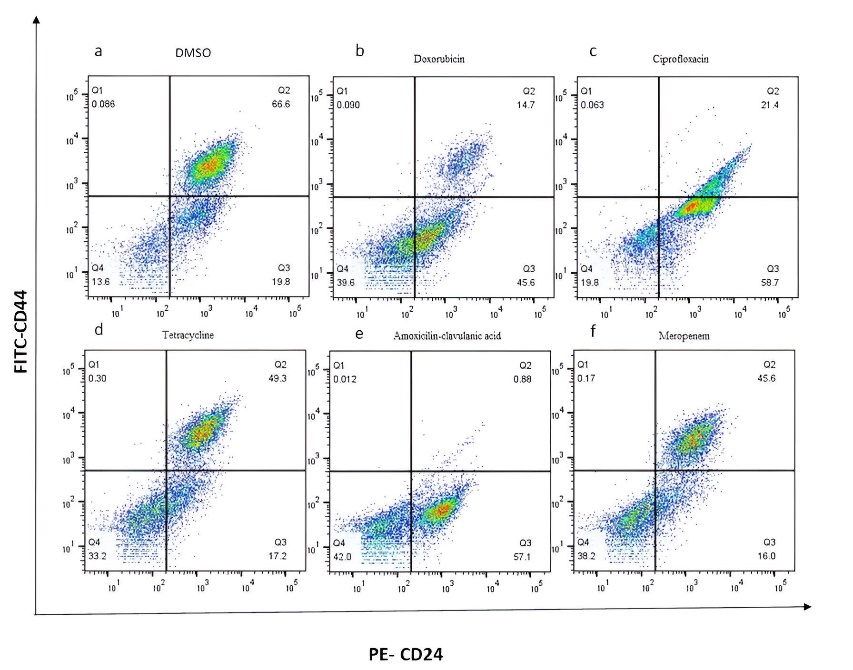


**Figure 5.** Effects of antibiotics on CD24/CD44 cancer stem cell populations in MDA-MB-468 breast cancer cells. a) DMSO b) doxorubicin, c) ciprofloxacin, d) tetracycline, e) amoxicillin-clavulanic acid, and f) meropenem. Q1: CD2high/CD44low, Q2: CD24high/CD44high, Q3: CD24low/CD44high, Q4: CD24low/CD44low populations

**Supplementary Figure 6 – “Doxorubicin-Antibiotics Treatments Show Higher on CD24/CD44 cancer stem cell population in MDA-MB-468 breast cancer cells”**


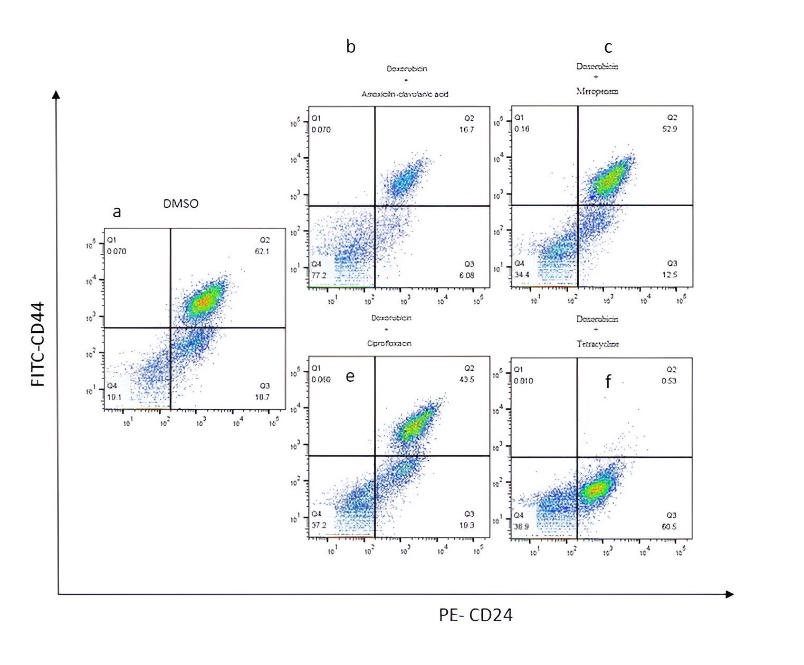


**Figure 6.** Effects of doxorubicin-antibiotics co-treatment on CD24/CD44 cancer stem cell population in MDA-MB-468 breast cancer cells. a) DMSO b) doxorubicin + amoxicillin-clavulanic acid, c) doxorubicin + tetracycline, d) doxorubicin + ciprofloxacin, and e) doxorubicin + meropenem Q1: CD24high/CD44low, Q2: CD24high/CD44high, Q3: CD24low/CD44high, Q4: CD24low/CD44low populations.
